# Integrative approach for differentially overexpressed genes in gastric cancer by combining large-scale gene expression profiling and network analysis

**DOI:** 10.1038/sj.bjc.6604682

**Published:** 2008-09-30

**Authors:** A Takeno, I Takemasa, Y Doki, M Yamasaki, H Miyata, S Takiguchi, Y Fujiwara, K Matsubara, M Monden

**Affiliations:** 1 Department of Surgery, Graduate School of Medicine, Osaka University, 2-2 Yamadaoka Suita, Osaka 565-0871, Japan; 2 DNA Chip Research Inc., 1-1-43 Suehirocho, Tsurumi, Yokohama, Kanagawa 230-0045, Japan

**Keywords:** gastric cancer, gene expression profiling, network mapping, focus genes, common gene activation, integrative approach

## Abstract

Gene expression profiling is a valuable tool for identifying differentially expressed genes in studies of disease subtype and patient outcome for various cancers. However, it remains difficult to assign biological significance to the vast number of genes. There is an increasing awareness of gene expression profile as an important part of the contextual molecular network at play in complex biological processes such as cancer initiation and progression. This study analysed the transcriptional profiles commonly activated at different stages of gastric cancers using an integrated approach combining gene expression profiling of 222 human tissues and gene regulatory dynamic mapping. We focused on an inferred core network with *CDKN1A* (*p21*^*WAF1/CIP1*^) as the hub, and extracted seven candidates for gastric carcinogenesis (*MMP7, SPARC, SOD2, INHBA, IGFBP7, NEK6, LUM*). They were classified into two groups based on the correlation between expression level and stage. The seven genes were commonly activated and their expression levels tended to increase as disease progressed. NEK6 and INHBA are particularly promising candidate genes overexpressed at the protein level, as confirmed by immunohistochemistry and western blotting. This integrated approach could help to identify candidate players in gastric carcinogenesis and progression. These genes are potential markers of gastric cancer regardless of stage.

Gastric cancer remains a major cause of cancer deaths worldwide despite early detection and curative surgery. Prognosis is favourable in early-stage disease with 5-year survival rates of 90% reported following gastrectomy and lymph node dissection. In contrast, patients diagnosed with advanced-stage cancer have 5-year survival rates of 20–30%, and the overall poor survival outcome for gastric cancer is attributed to these patient populations (Dicken *et al*, 2005). An efficient system for detecting disease status in gastric cancer regardless of its clinical stage is clearly needed to improve overall survival.

Gastric cancer is routinely classified according to the tumour-node-metastasis parameters of the primary tumour, lymph nodes, and metastasis. This classification helps the clinician to stage the tumour and develop a management strategy, as well as to provide an indication of prognosis. However, this conventional classification is not strong enough to predict individual prognosis, rendering uniform adjuvant therapy of limited value because of unnecessary adverse events. The use of molecular markers or gene profiling coupled with multivariate predictive models is designed to attain more accurate prognostic models. Recent molecular analyses revealed that gastric cancers closely associate with alterations in several interesting genes, such as p53 (Tamura *et al*, 1991; Uchino *et al*, 1993), p21 (Czerniak *et al*, 1989), c-met (Kaji *et al*, 1996), TGF-*β* (Park *et al*, 1994; Nakamura *et al*, 1998), and *β*-catenin (Park *et al*, 1999). However, these single candidate molecules yield different results among studies and the available data are unconvincing. Thus, the potential use of combinations of multiple markers instead of a single marker has been previously commented upon for the understanding of cancer biology or the prediction of patient prognosis (Lee *et al*, 2007).

The past decade has seen a revolution in high-throughput technologies for molecular profiling in cancer research. Particularly, gene expression profiling has enabled researchers to quantify biological states and consequently uncover subtle phenotypes important in cancer. Such analyses of tumour tissues have provided unique opportunities to develop profiles that can distinguish, identify, and classify discrete subsets of disease, predict the disease outcome, and even predict the response to therapy (Golub *et al*, 1999; Perou *et al*, 2000; van 't Veer *et al*, 2002; van de Vijver *et al*, 2002; Pittman *et al*, 2004). For example, expression profiling in gastric cancer identified novel target molecules involved in gastric carcinogenesis by comparing cancerous and healthy tissues (Boussioutas *et al*, 2003; Kim *et al*, 2003, 2005).

Despite their potential power, gene expression profiling has major limitations. Interpreting the significance of identified genes without any unifying biological theme can be difficult, makeshift, and dependent on the biologist's area of expertise. It is frequently challenging to understand a specific regulatory network involving enormous numbers of proteins. Furthermore, an approach that ignores biological cues may generate poor reproducibility among different studies of the same biological system. To overcome these analytical challenges, several recent studies have focused on phenotypic analysis of primary tumours using gene expression profiling, with a view to further understanding the roles of signalling pathways deregulated by the oncogenic process (Rhodes and Chinnaiyan, 2005; Rhodes *et al*, 2005).

This study sought to identify transcriptional profiles commonly activated across a wide range of stages in gastric cancer, as well as core networks in gastric carcinogenesis. It used an integrated approach combining gene expression profiling of over 200 human tissues with dynamic gene mapping. We identified seven candidates among the network that reflected essential transcriptional features of neoplastic transformation and progression, and validated these quantitatively by real-time reverse transcription (RT)–PCR. We also evaluated the expression of the encoded proteins in gastric cancer tissues by immunohistochemistry and western blotting, and identified novel potential markers for detecting gastric cancers.

## Materials and methods

### Tissue samples

Samples were obtained from 222 patients with gastric cancer who underwent curative resection at the following institutions: Osaka University Hospital, National Osaka Hospital, Osaka Medical Center for Cancer and Cardiovascular Diseases, Sakai Municipal Hospital, Toyonaka Municipal Hospital, Mino Municipal Hospital, NTT West Osaka Hospital, Kinki Central Hospital, Suita Municipal Hospital, and Kansai Rosai Hospital. None of the patients received chemotherapy or radiotherapy before surgery. Tissues were evaluated macroscopically and microscopically according to the general rules for gastric cancer study in surgery and pathology in Japan. All cancers showed a depth of invasion beyond the subserosa. The clinical and pathological features are listed in Table 1. All aspects of our study protocol were performed according to the ethical guidelines set by the committee of the three Ministries of the Japanese Government, and each subject provided informed consent.

### Extraction of RNA and quality assessment

The tumour specimens were cut into pieces (approximately 8 mm^3^) within 2 h after surgical resection and stored in RNAlater™ (Ambion, Austin, TX) at −80°C until use. Total RNA was purified from clinical samples using TRIzol reagent (Invitrogen, San Diego, CA, USA) according to the protocol supplied by the manufacturer. RNA integrity was assessed using an Agilent 2100 Bioanalyzer and RNA 6000 LabChip kits (Yokokawa Analytical Systems, Tokyo, Japan). Only high-quality RNAs with intact 18S and 28S sequences were used for the subsequent analysis. Fifteen RNA samples extracted from normal gastric epithelium were mixed as a reference control.

### Preparation of fluorescently labelled aRNA targets and hybridisation

Extracted RNA samples were amplified with T7 RNA polymerase using the Amino Allyl MessageAmp™ aRNA kit (Ambion) according to the protocol provided by the manufacturer. The quality of each Amino Allyl-aRNA sample was checked on the Agilent 2100 Bioanalyzer. Five *μ*g of control and experimental aRNA samples were labelled with Cy3 and Cy5, respectively, mixed, and then hybridised on an oligonucleotide microarray covering 30 000 human probes (AceGene Human 30K; DNA Chip Research and Hitachi Software Engineering Co, Yokohama, Japan). The experimental protocol is available at http://www.dna-chip.co.jp/thesis/AceGeneProtocol.pdf. The microarrays were scanned using a ScanArray 4000 (GSI Lumonics, Billerica, MA, USA).

### Analysis of microarray data

Signal values were calculated by DNASISArray software (Hitachi, Tokyo). Following background subtraction, data with low signal intensities were excluded from additional investigation. In each sample, the Cy5/Cy3 ratio values were log-transformed and globally equalised to remove deviation of the signal intensity between whole Cy3- and Cy5-fluorescence by subtracting the median of all log (Cy5/Cy3) values from each log (Cy5/Cy3) value. Supplementary information is available on our website (http://www.dna-chip.co.jp/).

### Network analysis

The Ingenuity Pathway (INGP) analysis was used to depict several networks in gastric cancer. The INGP software is a web-delivered application that enables biologists to discover, visualise, and explore therapeutically relevant networks significant to gene expression data sets. A detailed description of INGP analysis is available at Ingenuity Systems website (http//www.ingenuity.com). The average log_2_ expression values were used to calculate the fold change between gastric cancer and normal epithelium. The data set containing gene identifiers and their corresponding expression values were then uploaded into the INGP as a tab-delimited text file for analysis. Each gene identifier was mapped to its corresponding gene object in the Ingenuity Pathway Knowledge Base.

To understand how the genes identified by inferential statistics are related as focus genes, we uploaded the target genes into the Ingenuity Knowledge Base and generated several networks. On the basis of focus genes, new and expanded pathway maps, connections, and specific gene–gene interactions were inferred, functionally analysed, and used to build on the existing pathway knowledge base. To generate networks, the knowledge base was queried for interactions between focus genes and all other gene objects stored therein. The output, displayed graphically as nodes (genes) and edges (the biological relationship between the nodes), represented a significantly consistent number of biological pathways and functions implicated by the empirical data sets.

### RT reaction

Complementary DNAs (cDNAs) were generated with avian myeloblastosis virus reverse transcriptase (Promega, Madison, WI, USA) using the protocol recommended by the manufacturer. Briefly, 1 *μ*g of RNA was mixed with RT reagents including oligo-(dT)_15_ primer and incubated at 42°C for 15 min, followed by heating at 95°C for 5 min for enzyme inactivation.

### Quantitative RT–PCR with the LightCycler™

To validate the microaray data, quantitative PCR was performed using real-time PCR with a LightCycler (Idaho Tech, ID, USA). PCR reagents contained 1X LightCycler DNA Master SYBR Green I (Roche Diagnostics, Mannheim, Germany), 0.2 *μ*M of each primer, 3 mM MgCl_2_, and 2 *μ*l of cDNA template. PCR conditions were as follows: one cycle of denaturing at 95°C for 10 min, followed by 40 cycles of 95°C for 15 s, 62°C for 5 s, and 72°C for 10 s. The housekeeping gene glyceraldehyde-3-phosphate dehydrogenase (*GAPDH*) was amplified quantitatively at the same time to verify the integrity of RNA and to improve the diagnostic quality of the technique. The intensity of fluorescence was calculated at each cycle and a standard curve was constructed with 10-fold serial dilutions of cDNA obtained from the mixture of normal gastric epitheliums. The primer sequences for PCR amplification are listed below: *MMP7* forward primer, 5′ → GTCTCGGAGGAGATGCTCAC → 3′ and reverse, 3′ → GAGGAATGTCCCATACCC → 5′; *SPARC* forward primer, 5′ → CATTGACGGGTACCTCTCCC → 3′ and reverse, 3′ → CGATATCCTTCTGCTTGATGC → 5′; *INHBA* forward primer, 5′ → ATCATTGCTCCCTCTGGCTA → 3′ and reverse, 3′ → ACGATTTGAGGTTGGCAAAG → 5′; *IGFBP7* forward primer, 5′ → AAGTAACTGGCTGGGTGCTG → 3′ and reverse, 3′ → TATAGCTCGGCACCTTCACC → 5′; *NEK6* forward primer, 5′ → TGTCTGCTGTACGAGATGGC → 3′ and reverse, 3′ → GATGCACATGCTGACCAGTT → 5′; *LUM* forward primer, 5′ → GACATAAAGAGCTTCTGCAA → 3′ and reverse, 3′ → TTGTTCCAGGATACAGATATT → 5′; *SOD2* forward primer, 5′ → GCAAGGAACAACAGGCCTTA → 3′ and reverse, 3′ → CAGCATAACGATCGTGGTTT → 5′; *GAPDH* forward primer, 5′ → CAACTACATGGTTTACATGTTC → 3′ and reverse, 3′ → GCCAGTGGACTCCACGAC → 5′.

### Immunohistochemistry

Sections (3.5-*μ*m thick) were deparaffinised in xylene and rehydrated. They were subjected to immunohistochemical analysis using the avidin–biotin–peroxidase complex (ABC) method with a Vectastain ABC-peroxidase kit (Vector Laboratories, Burlingame, CA, USA). The tissue sections were incubated overnight with the primary antibodies; anti-human INHBA (Serotec, Oxford, UK; 1 : 300 dilution) and anti-human NEK6 (GeneTex, San Antonio, TX; 1 : 200 dilution), at 4°C. Negative control staining was performed with the use of normal mouse or goat IgG instead of the primary antibody, yielding negative results in all patients.

### Western blotting

Frozen tumour and noncancerous tissues were homogenised in 0.5 ml radioimmunoprecipitation assay buffer (25 mmol l^−1^ Tris (pH 7.4), 50 mmol l^−1^ NaCl, 0.5% sodium deoxycholate, 2% NP40, and 0.2% SDS) containing protease inhibitors (1 mmol l^−1^ phenylmethylsulfonyl fluoride, 10 *μ*g ml^−1^ aprotinin, and 10 *μ*g ml^−1^ leupeptin). The homogenate was centrifuged at 12 000 **g** for 20 min at 4°C. The resulting supernatant was collected and total protein concentration was determined using the Bradford protein assay (Bio-Rad, Hercules, CA, USA). Then, 100 *μ*g of the total protein was premixed with loading buffer (0.05 mol l^−1^ Tris-HCl (pH 6.8), 2% SDS, 0.2 mol l^−1^ n-mercaptoethanol, 10% glycerol, and 0.001% bromophenol blue), boiled for 5 min, and subjected to SDS–PAGE on 10% gels. Proteins were then transferred onto polyvinylidene difluoride membrane (Boehringer Mannheim) using a transblot apparatus in a buffer containing 0.02 mol l^−1^ Tris-HCl (pH 8.3), 0.2 mol l^−1^ glycine, and 20% methanol. After blocking in 10% skim milk, the membrane was incubated overnight with anti-human INHBA (1 : 200 dilution), anti-human NEK6 (Abgent, San Diego, CA, USA; 1 : 500 dilution) at 4°C, or anti-actin (Sigma-Aldrich, St Louis, MO, USA; 1 : 1000 dilution) for 1 h at room temperature. After three washes each for 10 min with TBS (0.02 mol l^−1^ Tris-HCl (pH 7.5) and 0.1 mol l^−1^ NaCl) containing 0.2% Tween 20, the filter was incubated with secondary antibody at 1 : 1000 dilution. The protein bands were detected using the enhanced chemiluminescence detection system (Amersham, Arlington Heights, IL, USA) according to the instructions supplied by the manufacturer.

## Results

### Analysis of microarray data

The gene expression profiles of 222 primary gastric cancers were analysed on a 30K oligonucleotide DNA microarray. Of the full gene sequences (29 638 expressed genes excluding control spots), 271 (0.9%) genes showed >1.5-fold change in differential expression in at least 100 samples. Among these 271 genes, 50 had been described previously in gastric cancers, whereas 187 genes were previously not described in gastric cancer and 34 genes were categorised into ESTs (expressed sequence tags).

### Network analysis

**Figure 1:**
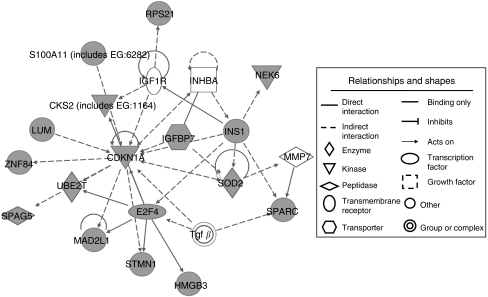
Inferential core network (network-5 and its close relevant networks) comprising many focus genes and several neighbourhood genes that connect the focus genes. Greyed nodes are part of network-5.

Analysis of the commonly overexpressed 271 genes using the Ingenuity Knowledge Base generated several networks that identified 203 genes as focus genes. The knowledge base generated 17 networks composed of focus genes and all other gene objects stored in the base (Table 2). On the basis of overlapping networks, network-5 was found to be central (Supplementary Figure 1). The centred network-5 (network-5 and close relevant networks) included a substantial number of genes already implicated in gastric carcinogenesis (Figure 1), with numerous focus genes connected by several neighbourhood genes. Furthermore, the network analysis mapped *CDKN1A* (*p21*^*WAF1/CIP1*^) to the core of the centred network-5, acting as a hub by interacting with surrounding focus genes. *CDKN1A* is associated with disease progression and prognosis in gastric cancer (Czerniak *et al*, 1989; Kasper *et al*, 1998).

We selected seven focus genes showing >2-fold change in differential expression for further analysis. Three of these are known to be involved in gastric cancer: *MMP7* (Yamashita *et al*, 1998), *SPARC* (Wang *et al*, 2004), and *SOD2* (Janssen *et al*, 2000), and the other four have no such reported associations (*INHBA, IGFBP7, NEK6,* and *LUM*).

### Correlation between activation of candidate gene and pathological stage

**Figure 2:**
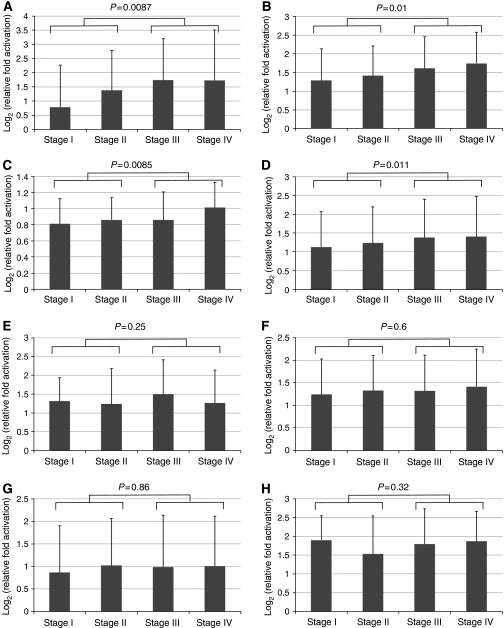
Correlation between activation of each candidate gene and pathological stage (*n*=222). The expression levels of genes of group 1 ((**A**) *MMP7*, (**B**) *IGFBP7*, (**C**) *NEK6*) correlated significantly with pathological stage (*P*=0.0087, 0.01, and 0.0085, respectively). The mean expression of the seven genes also correlated with pathological stage (*P*=0.011) (**D**). The expression levels of genes of group 2 ((**E**) *SOD2*, (**F**) *SPARC*, (**G**) *LUM*, (**H**) *INHBA*) did not correlate significantly with pathological stage (*P*=0.25, 0.6, 0.86, and 0.32, respectively).

To assess the clinical significance of each and common activation of the seven genes, we correlated microarray expression level and pathological stage. By comparing the expression level of each gene in early stage (stages I and II) and late stage (III and IV), we found that they could be classified into two groups: group 1 consisted of *MMP7*, *IGFBP7*, and *NEK6*; their expression levels correlated significantly with pathological stage (*P*=0.0087, 0.01, and 0.0085, respectively, Student's *t*-test) (Figure 2A–C), whereas the expression levels of genes of group 2 (*SOD2*, *SPARC*, *LUM*, *INHBA*) showed no such correlation (*P*=0.25, 0.6, 0.86 and 0.32, respectively, Student's *t*-test) (Figure 2E–H ). Interestingly, the mean expression of the seven genes correlated with the pathological stage (*P*=0.011) (Figure 2D).

### Validation of mRNA levels for selected genes using quantitative RT–PCR

**Figure 3:**
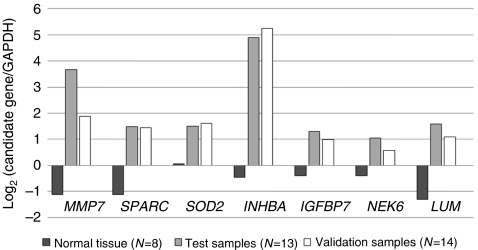
Bar chart shows mRNA levels of candidate genes using quantitative reverse transcription–PCR in normal gastric tissue (*n*=8, microarray reference control), test samples (*n*=13), and validation samples (*n*=14). Data are mean expression level of candidate gene relative to that of GAPDH in the examined tissues.

To provide further quantitative validation of our microarray data for the 7 genes, we analysed 13 test tumour samples by quantitative RT–PCR and compared the results with the quantified mRNA expression levels on the microarray (Figure 3). All 7 genes were highly expressed across the 13 cancers and the microarray data agreed with those obtained by quantitative RT–PCR. Similar agreement was found in a subsequent comparative analysis of 14 validation tumour samples (Figure 3). We also compared the expression of the candidate genes with the mean expression level of the corresponding genes in 8 normal tissues that were used for microarray reference control. The results showed upregulation of each candidate gene compared with that in the normal tissues (Figure 3).

### Protein expression of selected genes by immunohistochemistry and western blotting

**Figure 4:**
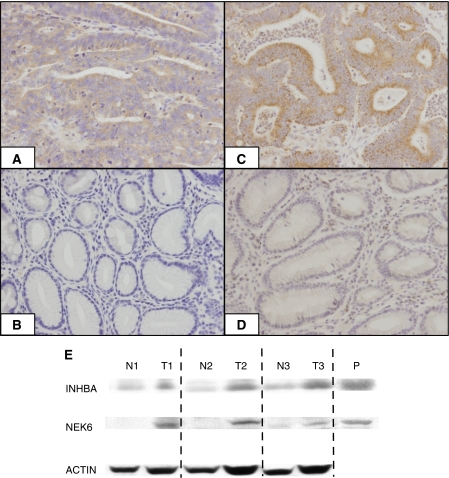
(**A**–**D**) Representative images of immunostaining for INHBA and NEK6. (**A**) Tumour tissue expressing INHBA; (**B**) healthy tissue for INHBA; (**C**) tumour tissue expressing NEK6; (**D**) healthy tissue for NEK6. Magnification, × 200. (**E**) Western blotting analysis of INHBA and NEK6 in three pairs of tumour (T) and normal (N) tissues. Anti-*β*-actin was used as control for protein level. P, positive control tissue.

Finally, we tested the encoded protein expression for each identified focus gene using immunohistochemistry and western blotting. Immunohistochemistry showed high expression of INHBA and NEK6 proteins in 14 of 20 and 24 of 27 tumour tissues, respectively (Figure 4A–D), whereas IGFBP7 and LUM proteins showed little immunoreactivity in tumour tissue relative to adjacent healthy tissue (data not shown). Each of these proteins was expressed in >50% cells in each tissue examined and all were localised into the cytoplasm.

Western blotting showed strong bands for both NEK6 and INHBA in gastric cancer tissues compared to normal tissue in all three pairs (Figure 4E).

## Discussion

Comprehensive gene expression profiling is a useful tool for analysing several thousands of genes in multiple samples simultaneously. In gastric cancer, this approach successfully discriminated cancerous and noncancerous tissues (Hippo *et al*, 2002). Since then, several studies have searched for novel genes related to carcinogenesis of gastric cancer and novel clinical subtypes related to biological malignancy using comprehensive gene expression profiling (Hasegawa *et al*, 2002; Ji *et al*, 2002; Boussioutas *et al*, 2003; Kim *et al*, 2003, 2005; Oien *et al*, 2003; Jinawath *et al*, 2004; Motoori *et al*, 2005). However, these data were generally obtained from human cell lines or small-scale tissue samples. Here, we analysed the gene expression profiles of more than 200 tissue samples covering every pathological stage, and verified the findings at both the mRNA and protein levels to increase the universality of our microarray data. Such a study is more likely to identify specific expression profiles that are commonly activated and thus more reflective of crucial transcriptional features of neoplastic transformation and progression in gastric cancers. In fact, increasing recognition that this large-scale, systematic approach is necessary to view the overall molecular events responsible for carcinogenesis has spawned several recent studies combining large-scale analysis of gene expression with knowledge-based and relevance network analysis (Bredel *et al*, 2005; Abdel-Aziz *et al*, 2007). Using such an approach also identified significantly upregulated genes linked to activated pathways as potential key molecules in hepatocellular carcinoma (Kittaka *et al*, 2008).

Dynamic mapping of 271 genes differentially expressed in gastric cancer tissues in this study revealed links among the majority of genes (203 genes, 84%) based on the Ingenuity Pathway Knowledge Base. This finding indicates that such gene populations do not act as individual units, but rather collaborate closely in overlapping networks during gastric carcinogenesis. Among the 17 networks identified here, network-5 was mapped to the centre of the overlapping network and contained the largest number of focus genes, implicating it as a key network. Furthermore, the identified networks assumed a cluster of robust genes implicated in gastric cancer-related genes. Our network analysis also revealed *CDKN1A (p21*^*WAF1/CIP1*^) as a hub gene that links to a large number of nodes and possibly determines the fundamental behaviour of the network.

The clinical significance of activation of our seven selected genes was further investigated by correlating the microarray expression data with the pathological stage. As indicated in Figure 2, we found these genes could be classified into two groups: the expression levels of genes of group 1 (*MMP7*, *IGFBP7*, and *NEK6*), but not those of group 2 (*SOD2, SPARC, LUM* and *INHBA*), correlated significantly with pathological stage. This finding indicates that although genes of group 2 may be involved in tumour formation and survival, those of group 1 may be involved in tumour progression. Their common activation seems to serve gastric carcinogenesis and tumour survival regardless of the pathological stage, based on the finding of overexpression of all seven genes in all samples. Furthermore, the gradual increase in the mean expression with cancer stage suggests that these genes cooperate in tumour progression. These results strengthen our proposal that such candidate genes are commonly activated during gastric carcinogenesis.

We also analysed the expression of the seven candidate genes based on age, sex, location, and histopathological type. Although the expression levels of *MMP7, NEK6, SOD2, SPARC,* and *INHBA* did not correlate with any of the above factors, *IGFBP7* and *LUM* were significantly upregulated in undifferentiated tumours compared to differentiated tumours (data not shown). These results suggest the involvement of these genes in tumour differentiation.

We also postulated that these genes are regulated by complex linkage between specific signalling pathways such as cell cycle signalling and TGF-*β* signalling, and that targeting several genes around *CDKN1A (p21*^*WAF1/CIP1*^), which functions as a hub, can compensate each other. The differential expressions were also corroborated by quantitative RT–PCR data in some of the previously tested tissue samples and in 14 validation samples. Together, these findings implicate all seven genes in gastric carcinogenesis, including the four that were not previously related to human gastric cancer.

Transcript profiling studies require complementary protein analysis to fully understand the associated regulatory process in living organisms. By itself, profiling does not adequately reflect the fluctuating signalling events occurring at the proteomic level, based on the evidence that only a subset of proteins correlate significantly with mRNA abundance (Chen *et al*, 2002; Nishizuka *et al*, 2003; Tian *et al*, 2004). These seemingly anomalous results are explained partly by translational processes whereby microRNAs repress the translation of mRNA into proteins, and partly by post-translational modifications such as phosphorylation, methylation, acetylation, and ubiquitination. For that reason, the expression levels of proteins encoded by highly overexpressed genes related to gastric carcinogenesis require further investigation. This study detected protein expression for two gene products among the four previously noncancer-related genes. Furthermore, NEK6 protein was strongly stained in most of the cancer tissues, but showed less mRNA signal compared to the remaining six genes. This finding suggests that NEK6 might be significantly modified post-translationally.

Matrix metalloproteinases including MMP7 play important roles in determining tumour invasion and metastasis and *MMP7* gene expression correlates with vessel invasion and both lymphatic and hematogenous metastases (Yamashita *et al*, 1998). Increased SPARC expression is linked to advanced gastric cancer (Wang *et al*, 2004), although the expression of SOD2 (Mn-SOD; manganese superoxide dismutase) was significantly enhanced in cancer tissues compared with normal mucosa, and the Mn-SOD ratio was proposed as an independent prognostic parameter (Janssen *et al*, 2000). The *IGFBP7* gene was upregulated in diffuse-type gastric cancer (Boussioutas *et al*, 2003) and in 22 gastric cancer/nontumour mucosa paired tissues samples (Kim *et al*, 2003). Interestingly, recent study revealed that TGF-*β* signalling including *INHBA* accounted for some of the main differences between normal tissue and gastric cancer at the transcript level (Yang *et al*, 2007).

As stated, this study identified several genes, such as LUM and NEK6, which were not previously associated with human gastric cancer. LUM is a member of the small leucine-rich proteoglycan family that induces apoptosis and suppresses cell proliferation. Its reduced expression has been associated with poor outcome in invasive carcinoma (Vuillermoz *et al*, 2004; Schuetz *et al*, 2006). NIMA (never in mitosis, gene A) was originally identified in *Aspergillus nidulans* as a serine/threonine kinase critical for cell cycle progression (Osmani *et al*, 1988). Human NIMA-related kinases (Neks) have high homology to NIMA in the N-terminal catalytic domain sequences. *NEK6* is a Neks-family gene required for mitotic progression in human cells (Roig *et al*, 2002). Inhibition of *NEK6* by either overexpression of an inactive *NEK6* mutant or elimination of endogenous *NEK6* using siRNA-arrested cells in M phase and triggered apoptosis (Belham *et al*, 2003; Yin *et al*, 2003). A recent study demonstrated overexpression of *NEK6* transcripts in hepatocellular carcinoma (Chen *et al*, 2006), although it was found to be frequently expressed among 125 serine/threonine kinase genes implicated in breast cancer, colorectal cancer, lung cancer, and laryngeal cancer by *in situ* hybridisation (Capra *et al*, 2006). However, no previous studies have shown *NEK6* expression in gastric cancers or NEK6 protein expression in any cancerous tissues. In data not shown here, we also found higher levels of NEK6 protein in advanced cancer compared to early-stage samples by immunohistochemistry.

In conclusion, this study used an integrated approach combining gene expression profiling and dynamic mapping of gene expression data on large sample numbers to identify novel candidate genes that may contribute to gastric carcinogenesis. The identified genes were universally validated in additional samples. In particular, NEK6 and INHBA are promising potential markers of gastric cancer regardless of disease stage.

## Figures and Tables

**Table 1:** Clinical and pathological features of 222 patients

Age (years) median (range)	68 (23–92)
Sex (male/female)	156 : 66
	
*Location*	
Upper	62
Middle	70
Lower	90
	
*Histopathological type*	
Differentiated	102
Undifferentiated	120
	
*Pathological stage*	
I	30
II	58
III	81
VI	53

**Table 2:** Seventeen networks identified in the data set

**ID**	**Molecules in network**	**Score**	**Focus molecules**
1	AEBP1, Ap1, APOC1, APOC2, APOE, BCL2A1, BID, CCL20, COX2, COX3, CTSL1, GDF15, GLA, HSPE1, IL1, IL32, INDO, LDL, LTA, LY96, MEOX2, MGP, MMP1, NCOR-LXR-Oxysterol-RXR-9 cis RA, NFκB, NR4A2, PDGF, Rar, RIPK2, Rxr, SERPINF1, SOD2, STK10, TNF receptor, TNFSF13B	45	26
2	Akt, COL1A1, CSE1 L, CXCL10, CYR61, FAP, FBN1, Fibrin, FN1, IFN-*γ*, Igfbp, IGFBP7, INHBA, Integrin, ITGB2, LTBP2, MIF, MMP, MMP7, MMP9, MMP12, PCOLCE, PI3K, PLAUR, PRKAA1, SLC3A2, SPARC, SULF1, TGFβ, TGFBI, THBS1, THY1, TIMP1, VCAN, VEGF	45	26
3	ACP5, ACTN1, ADORA3, AGXT, AIF1, CEBPB, CKS1B, CLEC4E, COL10A1, COL1A2, COL3A1, CREB, CREM, Cyclin A, Cyclin E, DNAJA1, E2f, ERK1/2, Histone h3, HLA-DPA1, HLA-DPB1, IFITM3, MAPK, MHC2*α*, PCNA, PKA, PTTG1, RFC4, SKP2, SPP1, STMN1, TGFBR1, UBE2C, Vitamin D3-VDR-RXR, ZNF160	43	25
4	C13ORF15, CACYBP, CDC2, CDKN3, COL4A1, FCER1G, FCGR2A, FCGR2B, FCGR3A, FOXM1, FPR1, GZMB, HOMER1, IGE, JNK, LAMA4, LAMB1, LAMC1, LGALS1, MAD2L1, MEK, MEK1/2, NFAT, P38 MAPK, PKC(s), PLA2G7, Rac, RAN, RANBP1, Ras, RGS1, Rsk, SRGN, TCR, UBD	41	24
5	Ap1, BUB3, CDKN1A, CKS1B, CKS2 (includes EG:1164), CLEC2B, E2F4, epinephrine, F9, fructose-2,6-diphosphate, GCNT1, HMCN1 (includes EG:83872), HMGB3, HSPE1, IGF1R, IL15, INS1, KIAA0101, LGALS3BP, LUM, MAD2L1, NEK6, NPHS2, PBK, PDCD5, RPS21, S100A11 (includes EG:6282), SOD2, SPAG5, SPARC, ST8SIA1, STMN1, UBE2T, VKORC1, ZNF84	31	20
6	Actin, ASB2, ATP6, ATP2B1, ATP5E, ATP6V1F, Caspase, CD163, Ck2, CLNS1A, F Actin, GEMIN5, H+-transporting two-sector ATPase, Insulin, JUB, LMNA, NEXN, PDGF BB, PFDN1, PFDN2, PFDN4, PFDN6, PLC, POLR2K, RNA polymerase II, RNU1B, S100A11 (includes EG:6282), SNRPD1, SNRPE, SNRPF, SNRPG, TCEB1, Ubiquitin, UCHL1, VBP1	27	18
7	ACP5, ARF4, BUB1 (includes EG:699), C1ORF164, C20ORF24, CCR6, CCT3, CCT4, CCT5, CCT7, CCT8, CCT6A, CPNE3, CTSB, CTSK, DAPK1, DEFB103A, EBNA1BP2, FCGR3A, FGFR, HTRA1, IFI30, IL4, IL10RA, ITGB7, keratan sulphate, MBP, MRPS10, MYL6, NAB2, NNMT, PRSS3 (includes EG:5646), TFF3, TGFB1, TUBA1A	27	18
8	CDK10, GBP4 (includes EG:115361), GPNMB, GPR109B, HLA-DPB1, HLA-DRA, IFI30, IFITM1, IFN*α*, IFN*β*, IFNAR1, IFNB1, IFNG, IFNK, ILF3, KIR2DL3, KIR2DS2, POMP, PTEN, PTP4A3, RARRES1, retinoic acid, RFX1, RFX5, RFXANK, RFXAP, RPS19, RPS20, SERPINA5, STX5, TBCB, TMSB10, TREM2, TREM3, TRIM22, TYROBP	25	17
9	CKLF, CLDN16, F2, GABRD, GGH, LAMP1, LAMP2, LEPRE1, MYC, MYCN, PAICS, PRDM5, Proteasome PA700/20s, PSMA, PSMA1, PSMA2, PSMA4, PSMA5, PSMA6, PSMA7, PSMB1, PSMB2, PSMB3, PSMB4, PSMB5, PSMB6, PSMB7, PSMD6, PSMD14, RPL31, RPL37, RPS19, RPS20, RPS27, TUBA1B	19	14
10	*β*-estradiol, BTK, dihydrotestosterone, EXOC1, EXOC2, EXOC3, EXOC5, EXOC7, EXOC8, FGF7, FGFR2, FSHB, GTP, GUCY1A3, INPP5F, ITGBL1, MLLT1, MME, NFE2L3, NME1, NME2, NUDT1, phosphatidylinositol-3,4,5-trisphosphate, PIB5PA, PRUNE, RAP1B, RND3, RRAD, SEC61G, SFRP2, SLC7A8, SLCO3A1, SPARC, TMEPAI, VHL	17	13
11	ABCB4, ACO1, ASXL1, ASXL2, ASXL3, C7ORF24, CBX2, CDKN2A, COL1A1, EZH2, FOS, FST, FTH1, FTL, FTMT, GAL, GNRHR, HIF1A, HIG2, iron, KNG1 (includes EG:3827), LOX, MELK, MYH4, NTF3, PCGF1, PCGF6, PCNX, PDGF Ab, PHF1, PHF19, progesterone, RPS6, SFRP4, TGFBI	12	10
12	ATP9B, Mg^2+^-ATPase	2	1
13	*N*-acetylglucosaminylphosphatidylinositol deacetylase, PIGL	2	1
14	Mrlc, MYLIP	2	1
15	EXOSC4, LRRC8D	2	1
16	GDP-Gnat2-Gngt2-Transducin *β* (cone), GNGT2, Gngt2-transducin beta (cone)	2	1
17	ADP-D-mannose, ADP-D-ribose, ADP-sugar diphosphatase, ADPribose diphosphatase, AMP, D-ribose-5-phosphate, nucleoside-diphosphatase, NUDT5	1	1
