# Supplementary figures and images for: Integrative approach for differentially overexpressed genes in gastric cancer by combining large-scale gene expression profiling and network analysis
Source: Br J Cancer. 2008 Sep 30;99(8):1307–15. doi: 10.1038/sj.bjc.6604682 (PMC2570518; doi:10.1038/sj.bjc.6604682)

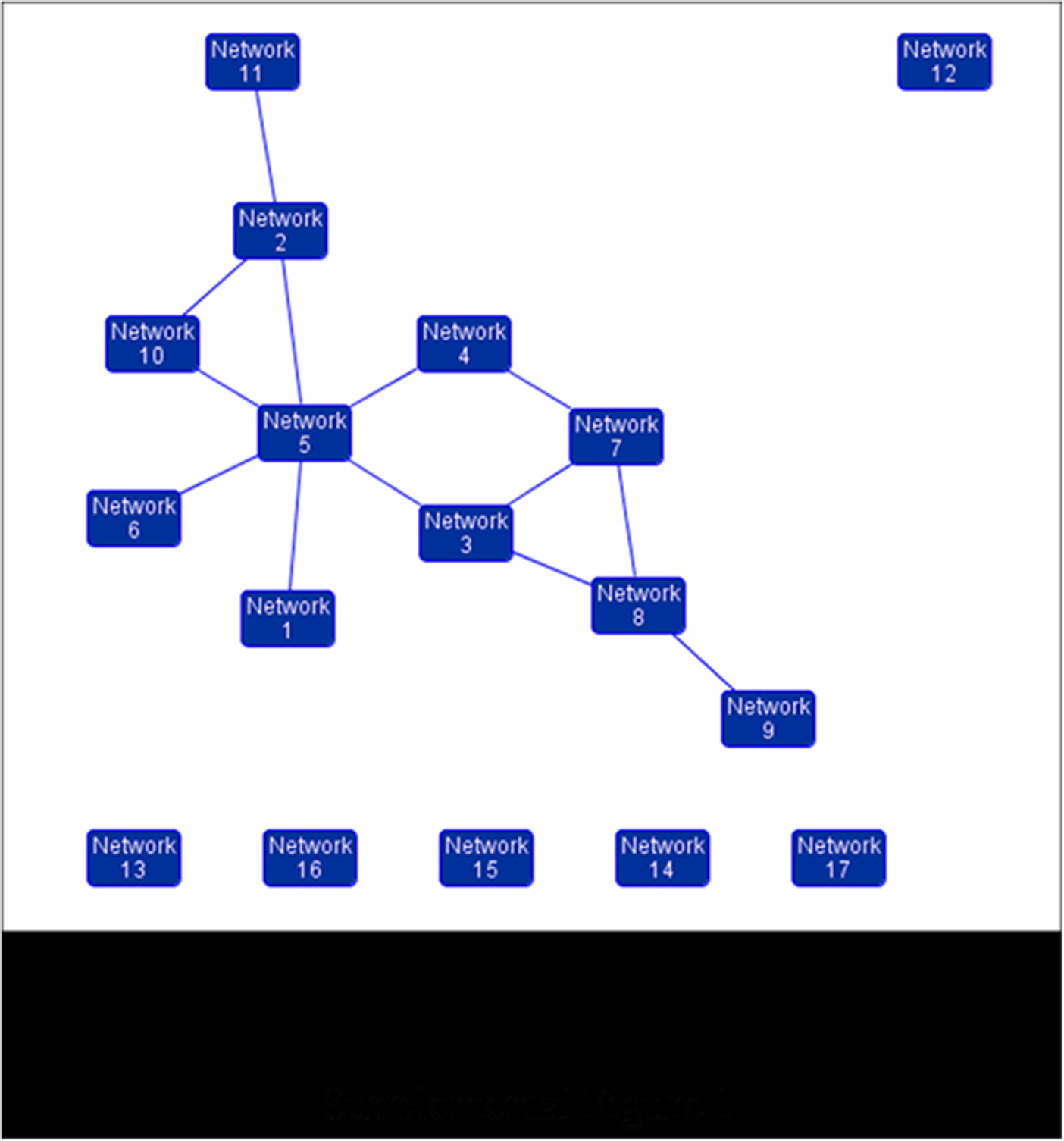

Supplement: Supplementary Figure 1 [file 6604682x1.tif]
