# Supplementary material for: Integrative approach for differentially overexpressed genes in gastric cancer by combining large-scale gene expression profiling and network analysis
Source: Br J Cancer. 2008 Sep 30;99(8):1307–15. doi: 10.1038/sj.bjc.6604682 (PMC2570518; doi:10.1038/sj.bjc.6604682)
Supplement: Supplementary Figure Legend [file 6604682x2.doc]

Supplemental figure: Overview of the overlapping 17 networks
